# Supplementary figures and images for: Sepsis recording in primary care electronic health records, linked hospital episodes and mortality records: Population-based cohort study in England
Source: PLoS One. 2020 Dec 31;15(12):e0244764. doi: 10.1371/journal.pone.0244764 (PMC7774940; doi:10.1371/journal.pone.0244764)

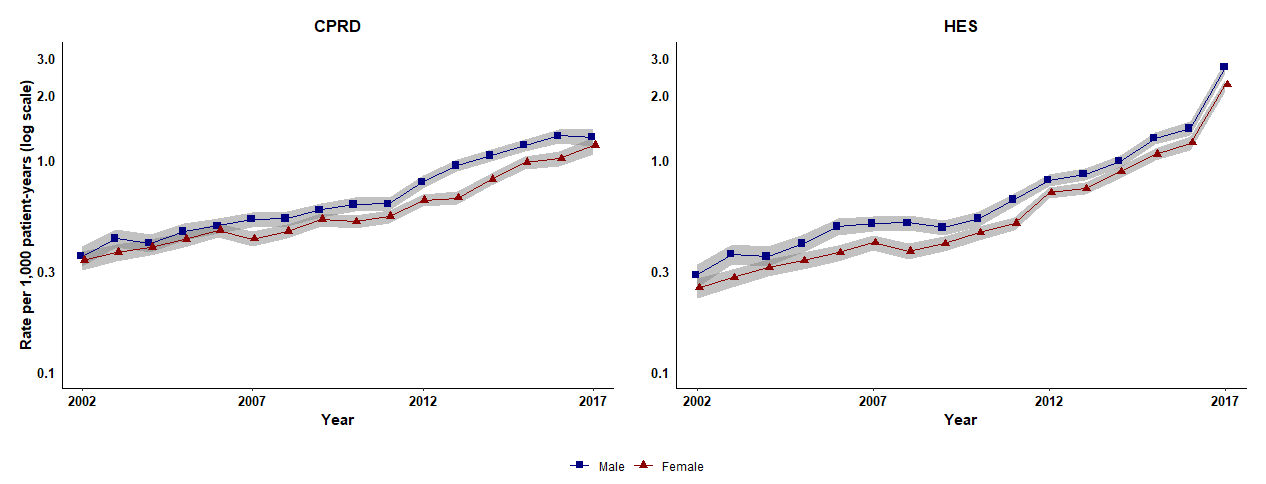

Supplement: S1 Fig — (TIF) [file pone.0244764.s002.tif]

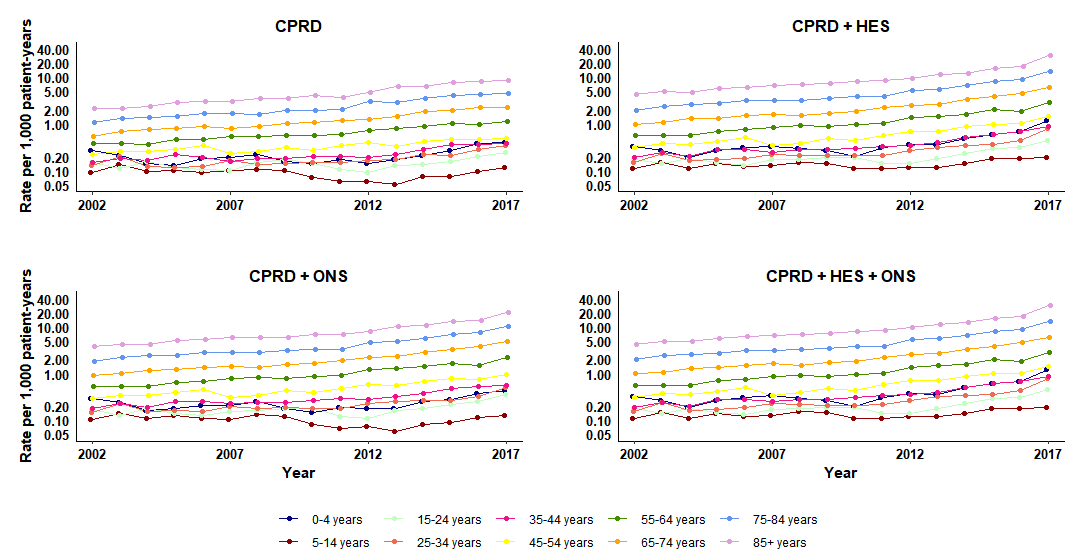

Supplement: S2 Fig — (TIF) [file pone.0244764.s003.tif]

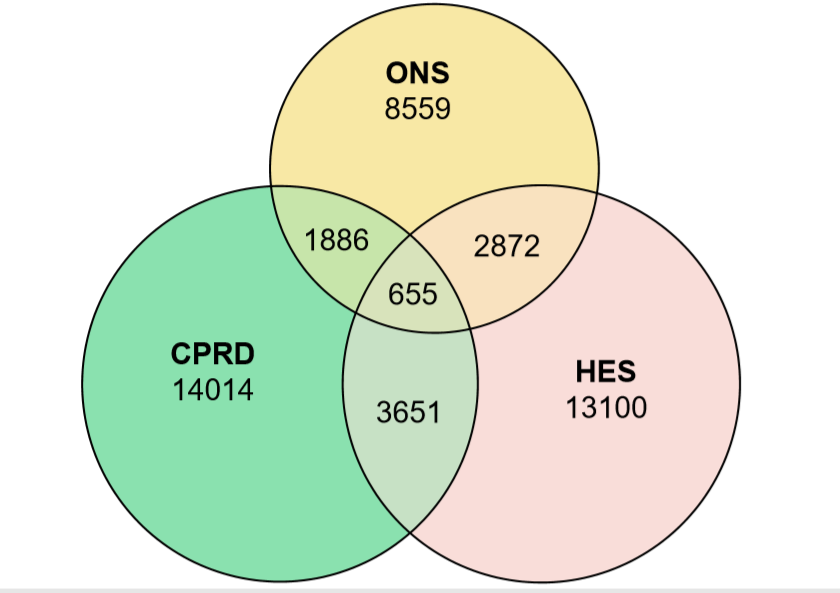

Supplement: S3 Fig — (TIF) [file pone.0244764.s004.tif]

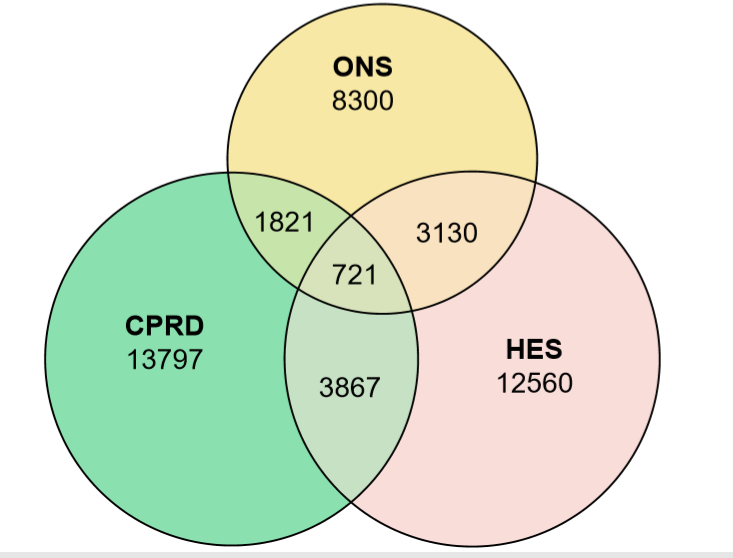

Supplement: S4 Fig — (TIF) [file pone.0244764.s005.tif]

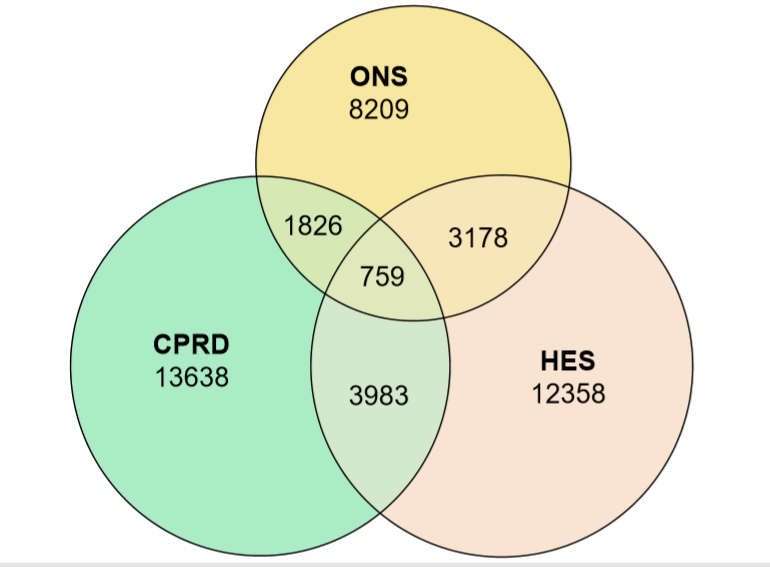

Supplement: S5 Fig — (TIF) [file pone.0244764.s006.tif]
