# Supplementary material for: Sepsis recording in primary care electronic health records, linked hospital episodes and mortality records: Population-based cohort study in England
Source: PLoS One. 2020 Dec 31;15(12):e0244764. doi: 10.1371/journal.pone.0244764 (PMC7774940; doi:10.1371/journal.pone.0244764)
Supplement: S1 Table — Figures are frequencies (% of column total). (DOCX) [file pone.0244764.s007.docx]

S1 Table. Concurrence of sepsis events in CPRD, ONS, HES using 30-day, 90- day and 150-day time-windows and either first or first and subsequent events. Figures are frequencies (% of column total).

|  | **CPRD** | **HES** | **ONS** |
| --- | --- | --- | --- |
|  |  |  |  |
| **First sepsis events** | 20 206 | 20 278 | 13 972 |
|  |  |  |  |
| **Concurrent first sepsis event associated with first primary care sepsis record** | | | |
| 30 days | - | 4 117 (20) | 2 438 (17) |
| 90 days | - | 4 588 (23) | 2 542 (18) |
| 150 days | - | 4 742 (23) | 2 585 (19) |
|  |  |  |  |
| **Concurrent first sepsis event associated with first HES sepsis record** | | | |
| 30 days | 4 117 (20) | - | 3 397 (24) |
| 90 days | 4 588 (23) | - | 3 851 (28) |
| 150 days | 4 742 (23) |  | 3 937 (28) |
|  |  |  |  |
| **Concurrent first or subsequent sepsis event following first primary care record** | | | |
| 30 days | - | 4 317 (21) | 2 541 (18) |
| 90 days | - | 4 770 (24) | 2 635 (19) |
| 150 days |  | 4 904 (24) | 2 669 (19) |
|  |  |  |  |
| **Concurrent first or subsequent sepsis event following first HES record** | | | |
| 30 days | 4 306 (21) | - | 3 527 (25) |
| 90 days | 4 710 (23) | - | 3 953 (28) |
| 150 days | 4 841 (24) |  | 4 025 (29) |
|  |  |  |  |

CPRD, Clinical Practice Research Datalink; HES, Hospital Episode Statistics; ONS, Office for National Statistics
